# Accelerated epigenetic aging and DNA methylation alterations in Berardinelli–Seip congenital lipodystrophy

**DOI:** 10.1093/hmg/ddad016

**Published:** 2023-01-28

**Authors:** Abeer Qannan, Yosra Bejaoui, Mahmoud Izadi, Noha A Yousri, Aleem Razzaq, Colette Christiansen, George M Martin, Jordana T Bell, Steve Horvath, Junko Oshima, Andre Megarbane, Johan Ericsson, Ehsan Pourkarimi, Nady El Hajj

**Affiliations:** College of Health and Life Sciences, Hamad Bin Khalifa University, Qatar Foundation, Doha 34110, Qatar; College of Health and Life Sciences, Hamad Bin Khalifa University, Qatar Foundation, Doha 34110, Qatar; College of Health and Life Sciences, Hamad Bin Khalifa University, Qatar Foundation, Doha 34110, Qatar; Genetic Medicine, Weill Cornell Medicine-Qatar, Doha, Qatar; Computer and Systems Engineering, Alexandria University, Alexandria, Egypt; College of Health and Life Sciences, Hamad Bin Khalifa University, Qatar Foundation, Doha 34110, Qatar; Department of Twin Research and Genetic Epidemiology, King’s College London, London, UK; Department of Laboratory Medicine and Pathology, University of Washington, Seattle, WA 98105, USA; Department of Twin Research and Genetic Epidemiology, King’s College London, London, UK; Altos Labs, San Diego, USA; Department of Human Genetics, David Geffen School of Medicine, University of California Los Angeles, Los Angeles, CA 90095, USA; Department of Laboratory Medicine and Pathology, University of Washington, Seattle, WA 98105, USA; Department of Clinical Cell Biology and Medicine, Graduate School of Medicine, Chiba University, Chiba, Japan; Department of Human Genetics, Gilbert and Rose-Marie Chagoury School of Medicine, Lebanese American University, Byblos, Lebanon; Institut Jérôme Lejeune, Paris, France; College of Health and Life Sciences, Hamad Bin Khalifa University, Qatar Foundation, Doha 34110, Qatar; College of Health and Life Sciences, Hamad Bin Khalifa University, Qatar Foundation, Doha 34110, Qatar; College of Health and Life Sciences, Hamad Bin Khalifa University, Qatar Foundation, Doha 34110, Qatar; College of Science and Engineering, Hamad Bin Khalifa University, Qatar Foundation, Doha 34110, Qatar

## Abstract

Berardinelli–Seip congenital lipodystrophy type 2 (CGL2) is a very rare human genetic disorder with potential significance to the understanding of the pathobiology of aging. CGL2 patients display characteristic progeroid features and suffer from type 2 diabetes, insulin resistance and fatty liver. In this study, we profiled genome-wide DNA methylation levels in CGL2 patients with *BSCL2* mutations to study epigenetic age acceleration and DNA methylation alterations. This analysis revealed significant age acceleration in blood DNA of CGL2 patients using both first- and second-generation epigenetic clocks. We also observed a shortened lifespan of *Caenorhabditis elegans* following knockdown of the *BSCL2* homolog *seip-1* on a *daf-16/*forkhead box, class O mutant background. DNA methylation analysis revealed significant differentially methylated sites enriched for lyase activity, kinase regulator activity, protein kinase regulator activity and kinase activator activity. We could also observe significant hypomethylation in the promoter of the dual specificity phosphatase 22 gene when comparing CGL2 patients versus controls. We conclude that in line with the observed progeroid features, CGL2 patients exhibit significant epigenetic age acceleration and DNA methylation alterations that might affect pathways/genes of potential relevance to the disease.

## Introduction

Lipodystrophies represent a wide spectrum of genetic or acquired disorders mainly characterized by perturbations in subcutaneous fat distribution and/or function (1–3). The abnormalities in fat tissue distribution can be either localized, partial or generalized. Congenital generalized lipodystrophies (CGLs) represent a heterogeneous group of recessive disorders where patients have a complete loss of body fat manifesting at birth (2,4,5). Patients have a striking muscular appearance due to the absence of subcutaneous fat and the increased fat storage in other organs, leading to muscular hypertrophy and organomegalies. Even though CGL patients are often nonobese, they suffer from severe metabolic complications, including insulin resistance, diabetes and hypertriglyceridemia (6,7). The frequency of the disease is reported to be around 1 in 12 million; however, this varies in certain countries and among different ethnic groups. For example, its incidence is estimated to be ~1 in 200 000 in Lebanon and ~1 in 25 000 in Oman due to the founder effect (8–10).

CGL is composed of four different subtypes caused by homozygous or compound heterozygous mutations in different genes. CGL1 is the most common type and is caused by pathogenic variants in the 1-acylglycerol-3-phosphate-O-acyltransferase 2 gene (11). Whereas mutations in the Berardinelli–Seip congenital lipodystrophy type 2 gene *BSCL2 *causes CGL2, the most severe form of the disease, also known as Berardinelli–Seip syndrome type 2 (12). Two additional subtypes affect a smaller number of patients, where mutations in the caveolin 1 and the polymerase I and transcript release factor *(PTRF)* genes have been reported in CGL3 and CGL4, respectively (13,14). The protein products of those four genes play important roles in the function of adipocytes and lipid droplet (LD) formation (15). *BSCL2* encodes seipin, an evolutionary conserved endoplasmic reticulum (ER) protein associated with LD biogenesis (16,17). In humans, seipin is highly expressed in adipose tissue, testis and brain (18). In the absence of seipin, lipid droplets (LDs) fail to grow leading to several tiny droplet s; however, those that grow develop into giant LDs due to changes in lipid synthesis. This effect has been observed in several model systems in response to perturbations in seipin (or its homologs) (16,19–25). It has been postulated that a major biological role of seipin is stabilizing membrane contact sites between LDs and the ER (17,24).

CGLs have been of interest in aging research since being proposed by Georges Martin in 1978 as a relevant genetic disorder for understanding the pathobiology of aging (26). Berardinelli–Seip syndrome is considered a segmental progeroid syndrome mainly because patients have a noticeable aged appearance as well as severe metabolic disturbances (27,28). Most patients have a life expectancy of <30 years, where death mainly occurs due to hypertrophic cardiomyopathy or hepatic failure secondary to hepatosteatosis (29). Recently, several studies reported epigenetic age acceleration in patients with progeroid features, including Werner syndrome (WS), Cockayne syndrome and Down’s syndrome (30–32). Furthermore, those patients were observed to have specific DNA methylation signatures affecting genes/pathways relevant to the associated clinical phenotypes (33,34). It is now well established that significant epigenetic alterations occur in response to aging in all cells and tissues across multiple species (35–37). To gain further insight into CGL2, we have performed the first genome-wide DNA methylation analysis in peripheral blood DNA of CGL2 patients to determine epigenetic age acceleration and identify differentially methylated regions associated with the disease. In addition, we looked at the effect of the *Caenorhabditis elegans* homolog for *BSCL2*, i.e. *seip-1,* on the worm’s lifespan.

## Results

### Epigenetic aging in Berardinelli–Seip syndrome

**Figure 1:**
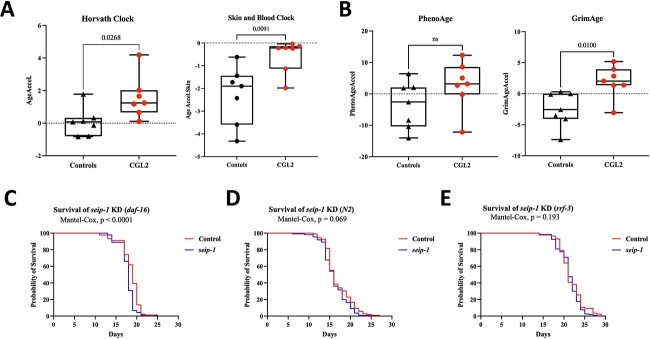
Epigenetic age acceleration in Berardinelli–Seip Congenital Lipodystrophy type 2 patients versus controls measured using (**A**) first-generation and (**B**) second-generation epigenetic clocks. Effect of seip-1 (ortholog of mammalian *BSCL2*) knockdown on the *C. elegans*’ lifespan in the (**C**) daf-16(mu86), (**D**) N2 (WT) worms and (**E**) RNAi hypersensitive mutant (rrf-3(pk1426)).

To establish whether *BSCL2* mutations are associated with age acceleration, we profiled genome-wide DNA methylation signatures in whole blood DNA of seven Berardinelli–Seip patients (average age = 5.85, range = 1–19) versus seven controls (average age = 7.85, range = 2–23), including samples from three healthy siblings who were homozygous for the wild type allele. We measured epigenetic age in blood DNA using widely used epigenetic clocks that measure age or human mortality risk. The calculated DNA methylation (DNAm) age had a strong linear relationship with chronological age in all samples and also in samples <20 years old, respectively (*r* = 0.99, *r* = 0.97) (Supplementary Material, Fig. S1a). This analysis revealed significant age acceleration in CGL2patients when compared with age and gender-matched controls using the Horvath pan tissue clock (*P* = 0.02), Skin and Blood Clock (*P* = 0.0091) and GrimAge (*P* = 0.01) clocks; however, the PhenoAge clock showed no significant differences in age acceleration (*P* = 0.14) (Fig. 1A and B). We additionally looked at intrinsic and extrinsic epigenetic age acceleration (IEAA and EEAA) between patients and controls. IEAA, which measures intrinsic age acceleration independent of blood cell proportions (38), revealed a significant increase in epigenetic age acceleration in patients with *BSCL2* mutations (*P* = 0.02). Whereas EEAA, which captures immune system aging, showed no significant differences (*P* = 0.11) (Supplementary Material, Fig. S2). We then regressed DNAm age on chronological age, disease status, gender, which revealed significant differences in DNAm age in CGL2 patients when compared with healthy controls (*P* = 2.04E-02, estimate = 1.65, SE = 0.60, Supplementary Material, Fig. S1b).

### Effect of *seip-1* knockdown on *C. elegans* lifespan

Next, we evaluated the effects of knocking down (KD) *seip-1* (*BSCL2* homolog) on the *C. elegans*’ lifespan in wild-type (N2) worms, the *daf-16,* and the RNA interference (RNAi) hypersensitive mutant, *rrf-3*. *daf-16* is the *C. elegans* counterpart of mammalian transcription factors of the forkhead box, class O (FoxO) and acts as a key regulator of longevity downstream of insulin and insulin-like growth factor signaling. Worms with the mutation in *daf-16* have a shorter lifespan compared with wild types. A survival analysis revealed that *seip-1* KD in the *daf-16* mutant significantly decreases the worm’s lifespan both in the Gehan–Breslow–Wilcoxon test and in the Log-rank (Mantel-Cox) test (*P*-value = 0.0001 and *P*-value < 0.0001, respectively) (Fig. 1C, Supplementary Material, Table S1). However, *seip-1* KD effect on the lifespan of the wild-type and *rrf-3* mutants was not significant with either Log-rank (Mantel-Cox) test (*P* = 0.069, *P* = 0.193, respectively) or the Gehan–Breslow–Wilcoxon test (*P*-value = 0.140, *P*-value = 0.697, respectively) (Fig. 1D and E, Supplementary Material, Tables S2 and S3).

### Differentially methylated CpG sites in congenital generalized lipodystrophy

**Figure 2:**
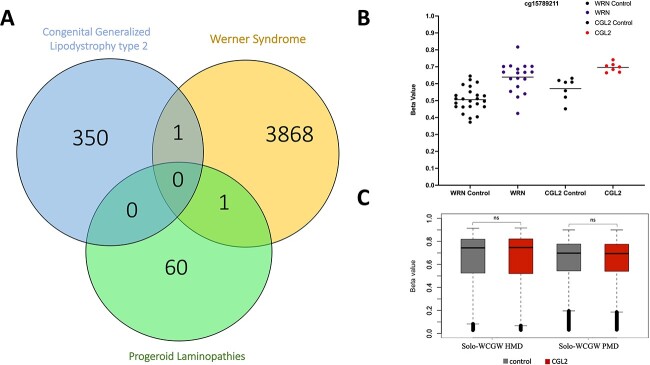
(**A**) Overlap between differentially methylated CpG sites in Berardinelli–Seip Congenital Lipodystrophy type 2, progeroid laminopathies, and typical Werner syndrome (WS). (**B**) DNA methylation levels in the overlapping cg15789211 site when comparing typical WS patients versus age- and gender-matched controls as well as CGL2 patients versus matched controls. (**C**) Solo-WCGW sites across HMDs and PMDs revealed no methylation difference between patients and controls.

We next compared genome-wide DNA methylation signatures in Berardinelli–Seip patients with *BSCL2* mutations versus matched controls. Differentially methylated sites/regions were analyzed after adjusting for age, gender, surrogate variables obtained by Surrogate Variable Analysis (SVA) and cell-type composition. The differential methylation analysis comparing seven Berardinelli–Seip patients versus seven controls revealed 351 significant differentially methylated sites with a false discovery rate (FDR) adjusted *P-*value < 0.05 (Supplementary Material, Table S4), including 94 CpG sites with an absolute difference in mean β > 0.1 (i.e. > 10% DNA methylation difference). Out of the 351 significant CpG sites, 186 were hypomethylated and 165 hypermethylated in CGL2 samples compared with controls. The significant CpG sites were enriched for several genes and were mostly located in intronic and intergenic regions (Supplementary Material, Fig. S3a and b). We next tested the enrichment of gene ontology (GO) sets and KEGG pathways on the 351 differentially methylated probes (DMPs)  using the methylGSA package, which performs the analysis after adjusting for the number of CpG sites per gene on the EPIC array. This revealed GO enrichment for lyase activity (adj. *P*-value = 2.88E-189), kinase regulator activity (adj. *P*-value = 2.28E-06), protein kinase regulator activity (adj. *P*-value = 2.28E-06), and kinase activator activity (adj. *P*-value = 5.51E-05) (Supplementary Material, Table S5). KEGG  (Kyoto Encyclopedia of Genes and Genomes) pathway analysis revealed that the DMPs were not associated with significant pathways following multiple testing corrections; nevertheless, the ‘Insulin signaling pathway’ was the highest ranked pathway (*P*-value = 0.01, adj. *P*-value = 0.35) (Supplementary Material, Table S6). We then applied the eFORGE tool to examine the overlap of DMPs with DNAse 1 hypersensitive sites (DHS) across multiple tissues using data from the Roadmap Epigenomics project. This revealed a clear significant overlap (*Q*-value < 0.01) with DHS sites in multiple fetal tissues, as well as in human embryonic stem cells (H1) and some blood cell types (Supplementary Material, Fig. S4). We further checked for overlap between the 351 DMPs in Berardinelli–Seip patients and differentially methylated CpG sites in progeroid laminopathies (GSE182991) and Werner syndrome (GSE131752) (33,39). This analysis revealed one common CpG site (cg06216080) with similar changes in blood DNA methylation of typical WS patients but not in progeroid laminopathy patients (Fig. 2A and B). Solo CpGs, which are located in a WCGW context in partially methylated domains (PMDs), lose methylation in response to cell proliferation (40). We performed an analysis of solo-WCGW sites across highly methylated domains (HMDs) and PMDs that revealed no methylation difference between patients and controls (*P*-value =0.60 and 0.22, respectively) (Fig. 2C).

### Differentially methylated regions in congenital generalized lipodystrophy

**Figure 3:**
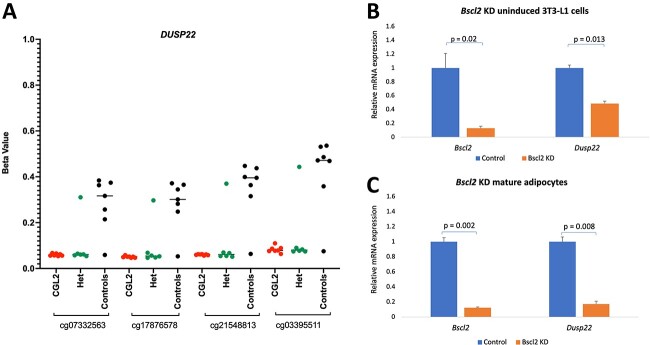
(**A**) DNA methylation levels of differentially methylated CpG sites located in the *DUSP22* promoter in CGL2 patients, *BSCL2* mutation carriers, and controls. *Dusp22* expression following *Bscl2* knockdown in (**B**) uninduced 3T3-L1 cells and (**C**) mature adipocytes.

We then performed an analysis focused on differentially methylated regions, including promoters, CpG islands and 5 kb tiling windows. Here, we observed 23 differentially methylated promoters between patients and controls (Supplementary Material, Table S7). However, only dual specificity phosphatase 22 (*DUSP22*) and *PM20D1* displayed significant differentially methylated promoters after filtering using a β methylation difference of >0.1 or <−0.1 (10% methylation difference) and >2 CpG sites in the region (Fig. 3A, Table 1, Supplementary Material, Fig. S5). At the CpG island level, we observed 24 differentially methylated regions; however, only the CGIs in the promoters of *DUSP22* and *PM20D1* were retained after applying the previously mentioned filtering criteria. Similarly, the tiling analysis (5 kb) revealed 12 differentially methylated regions, including two regions in *DUSP22* and *PM20D1,* after filtering. We next checked the effect of *BSCL2* mutations on *DUSP22* and *PM20D1* in the heterozygous healthy parents (carriers) of the Berardinelli–Seip congenital lipodystrophy patients. This revealed that all carriers apart from one individual had similar methylation differences to that observed in the CGL2 patients at the *DUSP22* promoter (Fig. 3A). The *PM20D1* region was described as methylation quantitative trait loci (mQTLs) where genetic variants could affect the DNA methylation profile of the CpG sites (41,42). For this reason, we mainly focused on the *DUSP22* promoter for further analysis, which has been reported as a negative mQTL region.

**Table 1:** Differentially methylated promoters in congenital generalized lipodystrophy type 2 patients with mean methylation difference >10% and >2 CpG sites

Gene	Chr	Start	End	Mean CGL2 patients	Mean controls	Beta methylation difference	Combined *P*-value	Combined FDR adjusted *P*-value	Number of CpG sites
*PM20D1*	chr1	205818761	205820760	0.30	0.42	−0.12	2.03E-07	0.00385059	12
*DUSP22*	chr6	290130	292129	0.07	0.30	−0.23	2.41E-06	0.0076298	4

Next, we checked two publicly available RNA sequencing (RNA-seq) datasets to investigate the effect of SEIPIN deficiency on *DUSP22* expression. In GSE159337, RNA-seq was performed on peripheral blood mononuclear cells of CGL2 patients and their age and gender-matched controls. However, this analysis revealed no significant change in the expression of *DUSP22* between the CGL2 patients and controls (adj. *P*-value = 0.71, log2FC = 0.12). Similarly, GSE145070 showed that adipocyte-specific conditional deletion of seipin in mouse mature brown adipocytes had no effect on *Dusp22* expression (adj. *P*-value = 0.99, log2FC = −0.02). Next, we looked at *DUSP22* and *BSCL2* gene expression changes during adipocyte differentiation. We observed a significant upregulation of *BSCL2* during early adipocyte differentiation (*P* < 0.01), but not during late differentiation (*P* = 0.123) (Supplementary Material, Fig. S6). For *DUSP22*, we did not detect significant differences following differentiation, nevertheless we could observe a tendency for transcriptional upregulation particularly during early differentiation (*P* = 0.08). Furthermore, we measured *Dusp22* mRNA expression after *Bscl2* knockdown in the murine 3 T3-L1 cell line, which is a commonly used *in vitro* model for white adipocytes.  A significant downregulation of *Dusp22* expression was observed in *Bscl2* knockdown cells compared with control cells in both uninduced (*P* = 0.013) and mature adipocytes (*P* = 0.008) (Fig. 3B and C). Thus, these data suggest that the two genes are linked and that *Bscl2* affects the expression of *Dusp22*.

### Effect of DNA methylation changes at *DUSP22* promoter

Here, we checked the association of DNA methylation levels at CpG sites located in the *DUSP22* promoters in adipose tissue for several metabolic phenotypes (BMI, visceral fat, insulin resistance, fasting blood glucose, incident T2D) in a 450 k methylation dataset from 538 female twins (43). We did not detect a significant association between *DUSP22* methylation levels and any of the investigated metabolic traits (Supplementary Material, Table S8). Similarly, we investigated whether blood DNA methylation at the *DUSP22* promoters is associated with body mass index (BMI),  low-density lipoprotein (LDL), high-density lipoprotein (HDL) and triglyceride in an EPIC methylation array dataset of 568 healthy controls. Here, we observed that DNA methylation of several CPG sites in the *DUSP22* promoter displayed significant association with HDL levels; however, this association was not significant following multiple testing correction (Supplementary Material, Table S9, Fig. S7).

## Discussion

In this study, we performed the first genome-wide DNA methylation analysis in CGL2 patients to determine epi-signatures and epigenetic alterations associated with the disease. CGLs are characterized by a near complete loss of normal adipose tissue and abnormal lipid buildup in other tissues, including liver, skeletal muscle and heart. CGL2 patients with biallelic null mutations in *BSCL2* suffer from early premature aging signs, including regional atrophy of subcutaneous tissues, as well as cardiovascular lesions, type 2 diabetes and psychomotor abnormalities. Nevertheless, Berardinelli–Seip congenital lipodystrophy has certain features discordant to normative aging, such as muscular hypertrophy, which is not surprising for a segmental progeroid syndrome (27,44). Our results confirm age acceleration at the molecular level, where we could observe increased epigenetic aging using a variety of epigenetic clocks, including the Horvath clock, Skin and Blood Clock, GrimAge and IEAA. The PhenoAge clock revealed no significant DNAm age acceleration; however, we could observe a tendency toward increased epigenetic age acceleration in the CGL type 2 patients. In contrast to IEAA, we did not observe differences in EEAA between patients and controls. IEAA is independent of blood cell composition and does not show differences due to confounded changes in blood cell counts. On the other hand, EEAA is based on the Hannum clock and is associated with age-dependent changes in blood cell counts, including naïve CD8+ T cells, exhausted CD8+ T cells and plasmablasts. Overall, we detected DNAm age acceleration using both the first-generation as well as the second-generation epigenetic clocks that are better trained to predict human mortality risk. This is in line with the effect observed in other diseases considered segmental progeroid syndromes, such as Werner syndrome and Cockayne syndrome, as well as Down syndrome, which is proposed by some researchers also to be a segmental progeroid syndrome (30,31,39). We observed no overlap when comparing CGL2 differentially methylated CpG sites to those identified in progeroid laminopathy patients, and only one common CpG site when compared with typical Werner syndrome. This indicates distinct DNA methylation alterations in response to mutations in *LMNA*, *WRN*, and *BSCL2*. Furthermore, progeroid laminopathies (including classic and non-classic Hutchinson-Gilford progeria syndrome) were reported not to exhibit epigenetic age acceleration as observed in Werner syndrome or CGL2, which suggests that accelerated aging in progeroid laminopathies is not related to the biological aging processes measured via epigenetic clocks (33). In this study, we also investigated the effect of KD seip-1/BSCL2 on the *C. elegans* lifespan. *Seip-1* RNAi did not affect the lifespans of wild-type and the RNAi hypersensitive mutants (*rrf-3*). However, KD *seip-1* in the *daf-16*/*FoxO* mutants reduced the lifespan. The shortening of the *daf-16* lifespan upon *seip-1* RNAi suggests that *daf-16* and *seip-1* have synthetic genetic interactions. In line with this observation, the insulin signaling pathway was the highest ranked pathway following KEGG enrichment analysis on DMPs in CGL2 patients.

*BSCL2* encodes a transmembrane protein (seipin) localized to the ER that has a role in adipocyte differentiation and controlling LD formation (45). LDs are intracellular lipid storage organelles integral to energy homeostasis as well as other processes, such as protein sequestration and membrane trafficking (46–48). During aging, damaged and misfolded proteins build up inside the cell and are ineffectively removed, thus impairing cellular function and tissue homeostasis (49,50). Misfolded proteins are removed using various pathways; however, recent data suggest initiating an LD-dependent backup system where LDs collect misfolded and damaged proteins from the ER (51,52). LDs also protect against lipid toxicity and oxidative stress by storing potentially toxic lipids (53,54). In recent years, the role of LDs in aging and age-related diseases is becoming more evident. Recently, Papsdorf *et al.* have reported a role for LDs and peroxisomes in promoting longevity mediated by dietary mono-unsaturated fatty acids (MUFAs) in *C. elegans*. The authors could also identify that an increase in the number of LDs, even in the absence of MUFAs, was similarly associated with a significant extension of lifespan (55). They induced MUFAs accumulation by increasing their production or decreasing their degradation via KD *ash-2* or *fat-2*, respectively. They proposed a critical role for seipin in the early stages of LD biogenesis by showing that increased MUFAs accumulation upon RNAi-induced KD of *ash-2* or *fat-2* did not increase *seip-1* mutants’ lifespan (55). In yeast, LD accumulation revealed no correlation with longevity, however, it was shown to protect aging cells against cold stress (56).

Till now, the mechanism via which SEIPIN deficiency inhibits adipogenesis, causing lipodystrophy remains unknown. Here, we could observe a significant difference at 351 significant CpG sites that were enriched for lyase activity, kinase regulator activity, protein kinase regulator activity and kinase activator activity. Lyases are a large group of enzymes, many of which are involved in lipid and glucose (glycolysis, the TCA cycle and gluconeogenesis) and lipid metabolism. One example is the mitochondrial 3-hydroxymethyl-3-methylglutaryl-coenzyme A (HMG-CoA) lyase. This enzyme catalyzes the cleavage of HMG-CoA into acetyl-CoA and acetoacetate, a key step in ketogenesis. HMG-CoA lyase deficiency is a rare genetic disorder associated with episodes of vomiting, diarrhea, dehydration, extreme tiredness, weak muscle tone and hypoglycemia. Another important member of this family is ATP-citrate lyase, which catalyzes the first step in fatty acid biosynthesis. Fructose-bisphosphate aldolase, also a lyase, catalyzes the reversible conversion of fructose 1,6-bisphosphate into dihydroxyacetone phosphate and glyceraldehyde 3-phosphate, thereby playing a key role in both glycolysis and gluconeogenesis. The eForge analysis revealed a discernable DHS enrichment pattern for those sites mainly in fetal tissues, which indicates a potential role for those epigenetic alterations in CGL patients mainly during development. Interestingly, we observed a strong hypomethylation of the *DUSP22* promoter in CGL2 patients as well as in *BSCL2* mutation carriers. *DUSP22* promoter was unmethylated in all CGL type 2 patients as well as in heterozygous mutation carriers apart from one individual that clustered with the controls. *DUSP22* is ubiquitously expressed in mammalian cells and is known to activate the cJun-N-terminal-kinase (JNK) signaling pathway (57). JNK activation is involved in obesity-induced insulin resistance as well as reduced compensatory insulin secretion response (58,59). *DUSP22* was reported as an obesity candidate gene hypermethylated in omental visceral adipose tissue but not in subcutaneous adipose tissue of obese subjects (60). Furthermore, it was shown to be differentially methylated in whole blood DNA when comparing high versus low responders to an intensive weight loss intervention where high responders had significantly lower *DUSP22* methylation at baseline prior to treatment (61). In addition, maternal obesity was also shown to be associated with lower methylation in *DUSP22* (including cg01516881) in children born to obese mothers (62). Our results revealed no association of *DUSP22* promoter methylation with several of the studied adiposity-related phenotypes in adipose tissue. We could also observe no association with BMI, LDL and triglyceride levels in peripheral blood DNA; however, a weak association with HDL levels was detected that did not survive multiple testing correction. The observed DNA methylation changes could be a likely contributor to CGL2 phenotypic characteristics or could occur as consequence of the effects of the condition. We could show an effect of *Bscl2* knockdown on *Dusp22* expression in mouse 3 T3-L1 cells. The same effect was not observed in blood of CGL2 patients or mature brown adipocytes with conditional seipin deletion. Nevertheless, further experiments are required to provide mechanistic insight into the effect of *DUSP22* and how SEIPIN deficiency affects its expression.

One of the limitations of this study is that we could only perform genome-wide DNA methylation analysis on a low number of CGL2 patients, which is inherently related to the low incidence rate of Berardinelli–Seip congenital lipodystrophy. Till now, only 250 patients have been reported in the literature, where CGL type 2 represents a fraction of those patients. Furthermore, we have only observed an effect of *seip-1* knockdown on reducing life span in a sensitive background of *daf-16*/*FoxO* mutants and not the wild-type *C. elegans*. It is of significant interest to study the effect of *seip-1* or human *BSCL2* on *C. elegans* life span. A gain of function mutation of *seip-1* or its overexpression can help to better define the genetic interaction between *BSCL2* and insulin signaling.

## Conclusion

Taken together, we could highlight a potentially important role for SEIPIN, a key component of the LD assembly protein complexes, in longevity and aging. We have observed epigenetic acceleration in CGL type 2 patients (with null mutations in *BSCL2)* as well as an effect of its homolog (*seip-1*) on modulating the *C. elegans*’ lifespan. Interestingly, we could also detect epigenetic alterations in response to seipin deficiency, including DNA methylation changes at the promoter of *DUSP22.* Nevertheless, our study only focused on CGL type 2; therefore, in future studies, it is important to check whether the observed epigenetic changes, as well as the epigenetic age acceleration, are specific to CGL type 2 or if it also occurs in CGL types 1, 3 and 4. This should provide a clearer understating of the effect of lipodystrophy as well as the protein machinery that controls LD formation in biological aging.

## Materials and Methods

### Study samples

Peripheral blood DNA from 7 CGL2 patients, ranging from 1 to 19 years of age, were collected, including five patients from three families in the community of the Mestizo tribe in the northern region of Peru with a homozygous p.Thr72Cysfs*2 mutation leading to the deletion of exon 3. Six individuals with heterozygous p.Thr72Cysfs*2 mutation were also recruited from these families. In addition, two samples from two unrelated Lebanese families with a homozygous p.Phe105fs*111mutation caused by a 5 bp deletion in exon 4 leading to a premature stop codon at position 111 were also included (12,44). Age and gender-matched controls, including three unaffected siblings, were selected as controls. The three related and four unrelated controls of European ethnicity (GSM5548210, GSM5548211, GSM5548212 and GSM5548216) were simultaneously processed with the patient samples. DNA methylation was measured using the Infinium Methylation EPIC Bead Chip microarray that covers over 850 000 CpG sites. The study was approved by the Institutional Review Board of the Qatar Biomedical Research Institute (QBRI) QBRI-IRB 2019-029 and the University of Washington FWA00006878, STUDY00000233.

### Genome-wide DNA methylation measurement using EPIC arrays

The EZ DNA Methylation Kit (Zymo Research, Irvine, CA, USA) was used for bisulfite conversion of ~500 ng of genomic DNA. Converted DNA was whole-genome amplified, enzymatically fragmented and hybridized to Infinium Methylation EPIC Bead Chips following the manufacturer’s protocol. To reduce the positional effects, cases and controls were randomly hybridized on the Infinium BeadChip arrays. Arrays were scanned via the Illumina iScan, and raw intensity data (IDAT) files were exported for analysis in R using the RnBeads package (63). Quality control and preprocessing steps included removing probes overlapping single nucleotide polymorphisms (SNPs), filtering out probes with the highest fraction of cross-hybridization and removing probes with the highest fraction of unreliable measurements via greedycut. Following preprocessing and quality control, 340 113 probes were removed and all samples were kept for further analysis. Furthermore, an additional stringent filtering step was performed via the ‘filtering.blacklist’ option to exclude Infinium MethylationEPIC probes with potentially polymorphic targets affected by SNPs, indels or structural variation in the following populations: European, African, admixed American, East Asian and South Asian. Next, Dasen (64) was used for data normalization and this step was followed by the removal of 17 633 probes on sex chromosomes. In total, 518 474 probes were kept for the differential DNA methylation analysis. White blood cell composition was estimated via the Houseman *et al.* (65) method. The comparison of deconvoluted cell proportions between cases and controls is presented in Supplementary Material, Figure S8.

### Differential DNA methylation and expression analysis

A limma-based analysis was performed to adjust for cell-type heterogeneity as well as other covariates, including age, gender and surrogate variables obtained by SVA. Differential methylation was performed at the following genomic regions: promoters, CpG islands, 5 kb tiling windows and genes. For each region, the mean difference in means for all CpG sites, the mean of quotients in mean methylation and the combined *P*-value for all CpG site *P*-values in the region were calculated. The FDR method was used to correct *P*-values for multiple testing. *DUSP22* gene expression analysis was performed in two publicly available GEO Datasets: 1) GSE159337, where RNA-seq was performed in 7 CGL2 patients compared with gender/aged-matched controls and 2) GSE145070, where RNA-seq transcriptome profiling was performed in brown adipose tissue from 10-week old *Bscl2* knockout mice with mature brown adipose tissue-specific deletion of *BSCL2* and 10-week old control mice (66,67). Furthermore, eFORGE, a web-based tool for interpreting EWAS data, was applied for functional overlap analysis for the chromatin-signal enrichment (68).

### Calculating DNA methylation age and age acceleration

Several epigenetic clocks utilizing different CpG sites were employed to estimate DNA methylation (DNAm) age. Pan-tissue (69), PhenoAge (70), GrimAge, (71) and SkinandBlood (72) clocks were used to measure DNAm age and age acceleration using the DNAm age calculator (https://dnamage.genetics.ucla.edu/) with the normalization option selected. In addition, IEAA and EEAA were measured.

### *C. elegans* strains and maintenance

*C. elegans* was maintained at 20°C and grown on Nematode Growth Medium (NGM) plates containing *Escherichia coli* (OP50 strain) as described previously (73). Strains used in this study: N2 Bristol (wild type); CF1038 *daf-16* (*mu86*) I; NL2090 *rrf-3*(*pk1426*) II.

### Knocking down *seip-1* using RNAi

The RNAi clone against *seip-1* (R01B10) from the Ahringer *C. elegans* RNAi library (Source Bioscience, UK) and the control RNAi bacterial strain (HT115) containing empty vector were used as described previously (74,75). In brief, bacteria expressing control RNAi (HT115), which contains an empty L4440 vector, and bacteria expressing *seip-1* RNAi were inoculated into 5 ml LB broth media supplemented with 100 mg/ml ampicillin and 10 mg/ml tetracycline and grown overnight at 37°C. The overnight bacterial cultures were regrown in 15 ml LB supplemented with 100 mg/ml ampicillin until they reached the optical density of 0.6–0.8. Bacterial cultures were pre-induced with 3 mM isopropyl-beta-D-thiogalactopyranoside (IPTG) for 1 h at room temperature. Bacterial cultures were seeded on the NGM plates containing 1 mM IPTG, 100 mg/ml ampicillin and 10 units/ml Nystatin.

### Survival assay

The survival assays were done as described previously (76) with minor modifications as following: To eliminate the possible maternal effect, P0 larva stage (L1) worms were treated with RNAi against *seip-1*. The highly synchronized F1 generation was collected by bleaching and was used for the survival assay. L1-staged worms were transferred to the matching RNAi plates seeded with either *seip-1* or the control RNAi (HT115). Once they reached to L4 stage, they were transferred to the RNAi plates containing 50 μM 5-fluorouracil (5-FU, ab142387, Abcam, UK). RNAi bacteria were added every 5–6 days to maintain the RNAi effect of the gene and avoid starvation. All experiments were repeated at least three times with an average of 100 worms per plate and scored with a dissecting microscope (M165C, Leica, Germany).

### Statistical analysis in *C. elegans*

A survival assay demonstrated the effect of KD *Seip-1* on worms’ life span. Kaplan–Meier survival curves were plotted using GraphPad Prism. Pairwise comparisons were conducted using the log-rank (*Mantel-Cox*) and *Gehan–Breslow–Wilcoxon* statistical tests. The ‘Mantel–Haenszel’ and ‘log-rank’ hazard ratios were calculated to test if the two interventions significantly interact. In contrast to the *Gehan–Breslow–Wilcoxon* method, which concentrates more on the subjects who died earlier, the log-rank test is more unbiased, gives the same importance to every time point and is used when the death rate is equal at all time points of the life span. Therefore, generalized Wilcoxon tests detect early changes between two survival curves, while the log-rank test is more sensitive to differences at later stages of life span (77).

### *DUSP22* methylation analysis in adipose tissue and peripheral blood DNA

DNA methylation levels were measured via the Infinium Methylation EPIC Bead Chip in 568 Qatari individuals enrolled at the Qatar BioBank. Linear regression models were used for the association analysis, correcting for age, gender, two principal components of actual cell counts (neutrophils, basophils, eosinophils, lymphocytes and monocytes), batch effect, plate number, well position, smoking surrogate (AHRR, cg05575921) and three genomic principal components for correction of population stratification.

### Cell culture and adipocyte differentiation

For this experiment, StemPro Human Adipose-Derived Stem Cells (Invitrogen) were used to measure the expression of *BSCL2* and *DUSP22* during differentiation at two separate time points. These cells are isolated from human lipoaspirate tissue and then cryopreserved after being expanded for one passage using MesenPRO RS medium. Differentiation of the fully confluent cells was induced with StemPro Adipogenesis Differentiation Kit (Invitrogen), while the control non-induced cells were maintained on MesenPRO RS Medium. After 1 week, multiple LDs started to develop, and the differentiation was stopped for one set of cells (first time point). For the later time point, a parallel set of cells were allowed to differentiate for 3 weeks, when mature adipocytes were obtained. Media was changed every 3 days and the cells were monitored by phase-contrast microscopy.

### *Bscl2* knockdown in the murine 3T3-L1 cell line

To knock down *Bscl2* in the murine 3 T3-L1 cell line, two lentiviral vectors containing short hairpin RNAs targeting Bscl2 were used (obtained from Vector Builder). A scrambled non-targeting lentiviral shRNA vector (pLKO.1-ctrl) was used as control. The shRNA vectors were co-transfected with lentiviral packaging plasmids to produce lentiviral particles, using calcium phosphate precipitation as described previously (78). 3T3L1 cells were transduced in regular media supplemented with polybrene (8 μg/ml). Twenty-four hours later, puromycin was added at 5 μg/ml for selecting the transduced cells. One set of cells were maintained in regular growth media, while another set of cells was induced for differentiation into mature adipocytes using differentiation medium I and II as described earlier (79). The efficiency of *Bscl2* knockdown and the expression of *Dusp22* were determined by quantitative PCR.

### Transcriptional profiling by real-time quantitative PCR

Total RNA was extracted from both sets of adipocytes according to the defined time points using PureLink RNA Micro Kit (Invitrogen). The RNA was reverse-transcribed using a High-Capacity cDNA Reverse Transcription Kit (Applied Biosystems) with random primers, according to the manufacturer’s protocol. Using PowerUp SYBR Green Master Mix (Applied Biosystems), the quantitative PCR (qPCR) reactions were optimized for the individual target genes using Quant Studio 6 Flex System. The expression of target genes was obtained in technical triplicates and normalized to the *HPRT/Hprt* housekeeping gene.

## Supplementary Material

Supplementary_Material_ddad016

Supplementary_Table_4_ddad016

## Data Availability

The IDAT files generated during this study are available in the Gene Expression Omnibus under accession number GSE214297.
