# Supplementary material for: Accelerated epigenetic aging and DNA methylation alterations in Berardinelli–Seip congenital lipodystrophy
Source: Hum Mol Genet. 2023 Jan 28;32(11):1826–35. doi: 10.1093/hmg/ddad016 (PMC10196674; doi:10.1093/hmg/ddad016)
Supplement: Supplementary_Material_ddad016 [file supplementary_material_ddad016.zip › Supplementary_Material_ddad016.docx]

| Comparison of Survival Curves for *daf-16* | | |
| --- | --- | --- |
|  |  |  |
| Log-rank (Mantel-Cox) test |  |  |
| Chi square | 16.33 |  |
| df | 1 |  |
| P value | <0.0001 |  |
| P value summary | **** |  |
| Are the survival curves sig different? | Yes |  |
|  |  |  |
| Gehan-Breslow-Wilcoxon test |  |  |
| Chi square | 14.86 |  |
| df | 1 |  |
| P value | 0.0001 |  |
| P value summary | *** |  |
| Are the survival curves sig different? | Yes |  |
|  |  |  |
| Median survival |  |  |
| HT115 | 19.00 |  |
| Seip-1 | 18.00 |  |
| Ratio (and its reciprocal) | 1.056 | 0.9474 |
| 95% CI of ratio | 0.7881 to 1.414 | 0.7073 to 1.269 |
|  |  |  |
| Hazard Ratio (Mantel-Haenszel) | A/B | B/A |
| Ratio (and its reciprocal) | 0.4485 | 2.230 |
| 95% CI of ratio | 0.3039 to 0.6617 | 1.511 to 3.290 |
|  |  |  |
| Hazard Ratio (logrank) | A/B | B/A |
| Ratio (and its reciprocal) | 0.6317 | 1.583 |
| 95% CI of ratio | 0.4684 to 0.8521 | 1.174 to 2.135 |

**Supplementary Table 1.** Comparison of Survival Curves for *daf-16*

| Comparison of Survival Curves for N2 | | |
| --- | --- | --- |
|  |  |  |
| Log-rank (Mantel-Cox) test |  |  |
| Chi square | 3.312 |  |
| df | 1 |  |
| P value | 0.0688 |  |
| P value summary | ns |  |
| Are the survival curves sig different? | No |  |
|  |  |  |
| Gehan-Breslow-Wilcoxon test |  |  |
| Chi square | 2.177 |  |
| df | 1 |  |
| P value | 0.1401 |  |
| P value summary | ns |  |
| Are the survival curves sig different? | No |  |
| Median survival |  |  |
| HT115 | 16.00 |  |
| Seip-1 | 16.00 |  |
| Ratio (and its reciprocal) | 1.000 | 1.000 |
| 95% CI of ratio | 0.7677 to 1.303 | 0.7677 to 1.303 |
| Hazard Ratio (Mantel-Haenszel) | A/B | B/A |
| Ratio (and its reciprocal) | 0.7505 | 1.332 |
| 95% CI of ratio | 0.5510 to 1.022 | 0.9781 to 1.815 |
| Hazard Ratio (logrank) | A/B | B/A |
| Ratio (and its reciprocal) | 0.8103 | 1.234 |
| 95% CI of ratio | 0.6213 to 1.057 | 0.9464 to 1.609 |

**Supplementary Table 2** Comparison of Survival Curves for N2

| Comparison of Survival Curves for rrf-3 | | |
| --- | --- | --- |
|  |  |  |
| Log-rank (Mantel-Cox) test |  |  |
| Chi square | 1.696 |  |
| df | 1 |  |
| P value | 0.1928 |  |
| P value summary | ns |  |
| Are the survival curves sig different? | No |  |
|  |  |  |
| Gehan-Breslow-Wilcoxon test |  |  |
| Chi square | 0.1521 |  |
| df | 1 |  |
| P value | 0.6966 |  |
| P value summary | ns |  |
| Are the survival curves sig different? | No |  |
|  |  |  |
| Median survival |  |  |
| HT115 | 21.00 |  |
| Seip-1 | 21.00 |  |
| Ratio (and its reciprocal) | 1.000 | 1.000 |
| 95% CI of ratio | 0.7581 to 1.319 | 0.7581 to 1.319 |
|  |  |  |
| Hazard Ratio (Mantel-Haenszel) | A/B | B/A |
| Ratio (and its reciprocal) | 0.8030 | 1.245 |
| 95% CI of ratio | 0.5772 to 1.117 | 0.8951 to 1.733 |
|  |  |  |
| Hazard Ratio (logrank) | A/B | B/A |
| Ratio (and its reciprocal) | 0.8584 | 1.165 |
| 95% CI of ratio | 0.6525 to 1.129 | 0.8855 to 1.533 |

**Supplementary Table 3** Comparison of Survival Curves for *rrf-3*

| **ID** | **Description** | **Size** | **p-value** | **p-adj** |
| --- | --- | --- | --- | --- |
| GO:0016829 | lyase activity | 292 | 2.19E-192 | 2.88E-189 |
| GO:0019207 | kinase regulator activity | 256 | 5.20E-09 | 2.28E-06 |
| GO:0019887 | protein kinase regulator activity | 222 | 5.20E-09 | 2.28E-06 |
| GO:0019209 | kinase activator activity | 100 | 1.67E-07 | 5.51E-05 |

**Suppmenentary Table 5** Gene ontology enrichment analysis for the 351 differentially methylated probes in CGL2 patients after adjusting for probe bias distribution in the Illumina Epic arrays.

| **ID** | **Description** | **Size** | **p-value** | **p-adj** |
| --- | --- | --- | --- | --- |
| 4910 | Insulin signaling pathway | 138 | 0.01 | 0.35 |
| 4660 | T cell receptor signaling pathway | 108 | 0.02 | 0.35 |
| 4270 | Vascular smooth muscle contraction | 116 | 0.10 | 0.68 |
| 230 | Purine metabolism | 162 | 0.15 | 0.68 |
| 4722 | Neurotrophin signaling pathway | 127 | 0.16 | 0.68 |
| 5145 | Toxoplasmosis | 132 | 0.16 | 0.68 |
| 5160 | Hepatitis C | 134 | 0.16 | 0.68 |
| 4650 | Natural killer cell mediated cytotoxicity | 136 | 0.26 | 0.79 |
| 4670 | Leukocyte transendothelial migration | 116 | 0.26 | 0.79 |
| 4810 | Regulation of actin cytoskeleton | 213 | 0.26 | 0.79 |

**Supplementary Table 6** KEGG pathway analysis for the 351 differentially methylated probes in CGL2 patients after adjusting for the number of CpG sites per gene on the Illumina Epic arrays.

| id | CHR | Start | End | symbol | Beta Methylation Difference | Combined FDR adjusted p-value | Number of CpG sites |
| --- | --- | --- | --- | --- | --- | --- | --- |
| ENSG00000264468 | chr17 | 6558329 | 6560328 | *MIR4520A;*  *MIR4520B* | 0.036 | 0.0024 | 4 |
| ENSG00000162877 | chr1 | 205818761 | 205820760 | *PM20D1* | -0.120 | 0.0039 | 12 |
| ENSG00000249287 | chr5 | 180397263 | 180399262 | *NA* | 0.083 | 0.0044 | 1 |
| ENSG00000261996 | chr17 | 6557829 | 6559828 | *NA* | 0.030 | 0.0044 | 5 |
| ENSG00000231228 | chr5 | 180407743 | 180409742 | *NA* | 0.057 | 0.0052 | 2 |
| ENSG00000233980 | chr7 | 76099876 | 76101875 | *FDPSP2* | -0.013 | 0.0052 | 1 |
| ENSG00000241850 | chr22 | 24346759 | 24348758 | *GSTTP1* | -0.080 | 0.0052 | 1 |
| ENSG00000229513 | chr22 | 24346940 | 24348939 | *EIF4EBP1P2* | -0.080 | 0.0052 | 1 |
| ENSG00000253299 | chr8 | 101177207 | 101179206 | *NA* | 0.210 | 0.0055 | 1 |
| ENSG00000215559 | chr21 | 15352259 | 15354258 | *ANKRD20A11P* | 0.009 | 0.0055 | 2 |
| ENSG00000270123 | chr5 | 135415787 | 135417786 | *VTRNA2-1* | 0.040 | 0.0060 | 9 |
| ENSG00000112679 | chr6 | 290130 | 292129 | *DUSP22* | -0.231 | 0.0076 | 4 |
| ENSG00000222179 | chr18 | 43569412 | 43571411 | *RN7SKP26* | -0.154 | 0.0104 | 1 |
| ENSG00000259038 | chr14 | 69094663 | 69096662 | *NA* | 0.048 | 0.0115 | 5 |
| ENSG00000242375 | chr9 | 99959470 | 99961469 | *NA* | -0.106 | 0.0143 | 1 |
| ENSG00000262026 | chr16 | 30788270 | 30790269 | *NA* | -0.150 | 0.0143 | 1 |
| ENSG00000229644 | chr10 | 36812663 | 36814662 | *NAMPTL* | -0.004 | 0.0157 | 1 |
| ENSG00000236874 | chr20 | 47013139 | 47015138 | *NA* | -0.004 | 0.0157 | 1 |
| ENSG00000231496 | chr10 | 11000614 | 11002613 | *NA* | -0.015 | 0.0205 | 1 |
| ENSG00000248903 | chr11 | 71278226 | 71280225 | *NA* | -0.214 | 0.0274 | 1 |
| ENSG00000250312 | chr4 | 122886 | 124885 | *ZNF718* | -0.065 | 0.0430 | 6 |
| ENSG00000140950 | chr16 | 84587140 | 84589139 | *TLDC1* | 0.065 | 0.0430 | 1 |
| ENSG00000168903 | chr5 | 180414345 | 180416344 | *BTNL3* | 0.044 | 0.0431 | 1 |

**Supplementary Table 7** Differentially methylated promoters between CGL2 patients and controls

| **IlmnID** | **CHR** | **MAPINFO** | **Methyl 450 Loci** | **BMI**  **p-value** | **VF (with BMI covariate)**  **p-value** | **IR**  **p-value** | **iT2D**  **p-value** | **FBG**  **p-value** |
| --- | --- | --- | --- | --- | --- | --- | --- | --- |
| **cg04267224** | 6 | 290588 | False | N/A | N/A | N/A | N/A | N/A |
| **cg07332563** | 6 | 291687 | TRUE | 0.55496 | 0.24451 | 0.50522 | 0.32935 | 0.58816 |
| **cg17876578** | 6 | 291859 | False | N/A | N/A | N/A | N/A | N/A |
| **cg21548813** | 6 | 291882 | TRUE | 0.05837 | 0.05481 | 0.97467 | 0.3969 | 0.15708 |
| **cg03395511** | 6 | 291903 | TRUE | 0.66978 | 0.34077 | 0.76283 | 0.12797 | 0.19489 |
| **cg18110333** | 6 | 292329 | TRUE | 0.71705 | 0.25807 | 0.41041 | 0.06031 | 0.09856 |
| **cg05064044** | 6 | 292385 | TRUE | 0.05004 | 0.13782 | 0.94121 | 0.82123 | 0.2003 |
| **cg11235426** | 6 | 292522 | TRUE | 0.73174 | 0.4536 | 0.48412 | 0.68311 | 0.16493 |
| **cg01516881** | 6 | 292596 | TRUE | 0.55336 | 0.51338 | 0.4619 | 0.25812 | 0.38107 |
| **cg26668828** | 6 | 292823 | TRUE | 0.87243 | 0.76751 | 0.97002 | 0.51945 | 0.64901 |
| **cg01171360** | 6 | 293285 | TRUE | 0.73565 | 0.42269 | 0.60008 | 0.50847 | 0.84886 |

**Supplementary Table 8** DNA methylation association of CpG sites in the *DUSP22* promoter with BMI, visceral fat, insulin resistance, fasting blood glucose, incident T2D in a 450k methylation dataset of 538 female twins.

| **IlmnID** | **CHR** | **MAPINFO** | **HDL p-value** |
| --- | --- | --- | --- |
| cg04267224 | 6 | 290588 | 0.08152866 |
| cg07332563 | 6 | 291687 | 0.03561572 |
| cg17876578 | 6 | 291859 | 0.02715611 |
| cg21548813 | 6 | 291882 | 0.03538749 |
| cg03395511 | 6 | 291903 | 0.04300991 |
| cg18110333 | 6 | 292329 | 0.02749478 |
| cg05064044 | 6 | 292385 | 0.0218576 |
| cg11235426 | 6 | 292522 | 0.05884858 |
| cg01516881 | 6 | 292596 | 0.05377964 |
| cg26668828 | 6 | 292823 | 0.09442684 |
| cg01171360 | 6 | 293285 | 0.10664074 |

**Supplementary Table 9** DNA methylation association of CpG sites in the *DUSP22* promoter with HDL in an EPIC methylation array dataset of 568 healthy controls from the Qatar BioBank.

**Supplementary Figures**

**
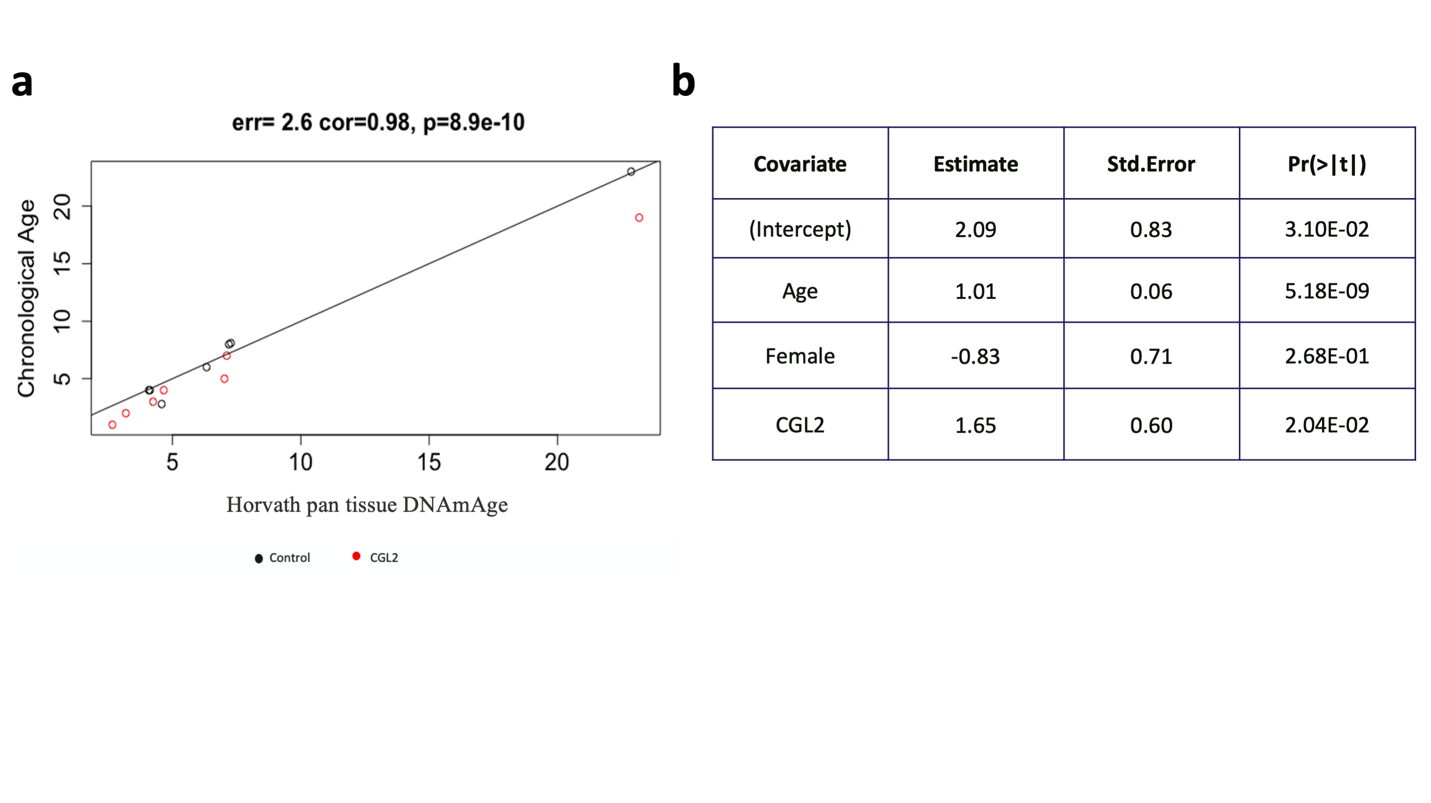
**

**Supplementary Figure 1 a.** Correlation analysis of chronological age vs DNA methylation age **b.** Linear regression analysis of DNAm age on chronological age, gender, and disease status.

**Supplemetary Figure** **2 a.** Intrinsic (IEAA) and **b.** extrinsic epigenetic age acceleration (EEAA) in Berardinelli–Seip Congenital Lipodystrophy (type 2) vs controls.


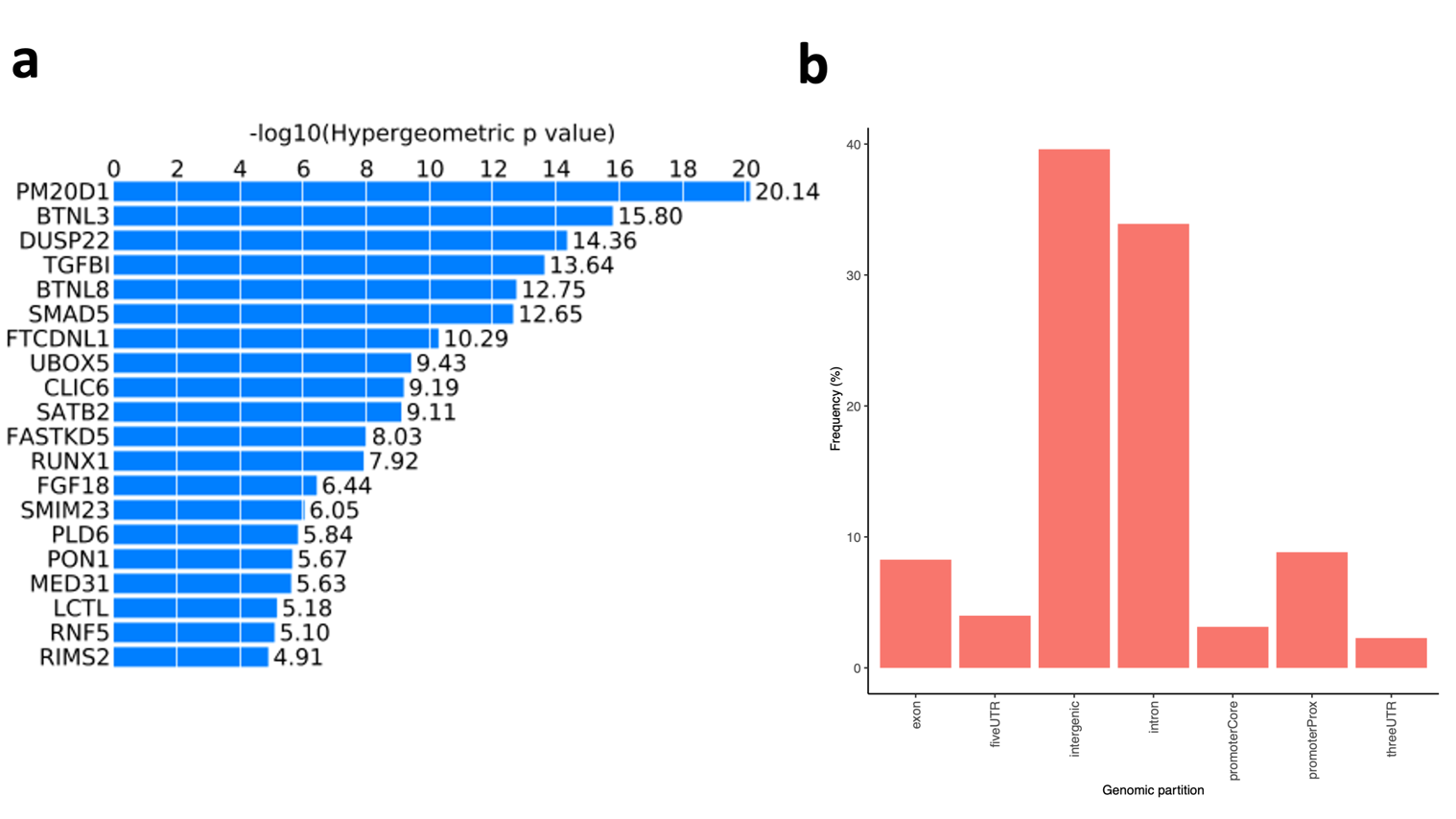


**Supplementary Figure 3 a.** Enriched genes in the list of 351 significant DMPs and **b.** distribution of DMPs across various genomic features.

**Supplementary Figure 4.** eFORGE analysis to test for cell type or tissue specific signal between DNAse 1 hypersensitive sites and differentially methylated CpG sites in CGL2 patients.


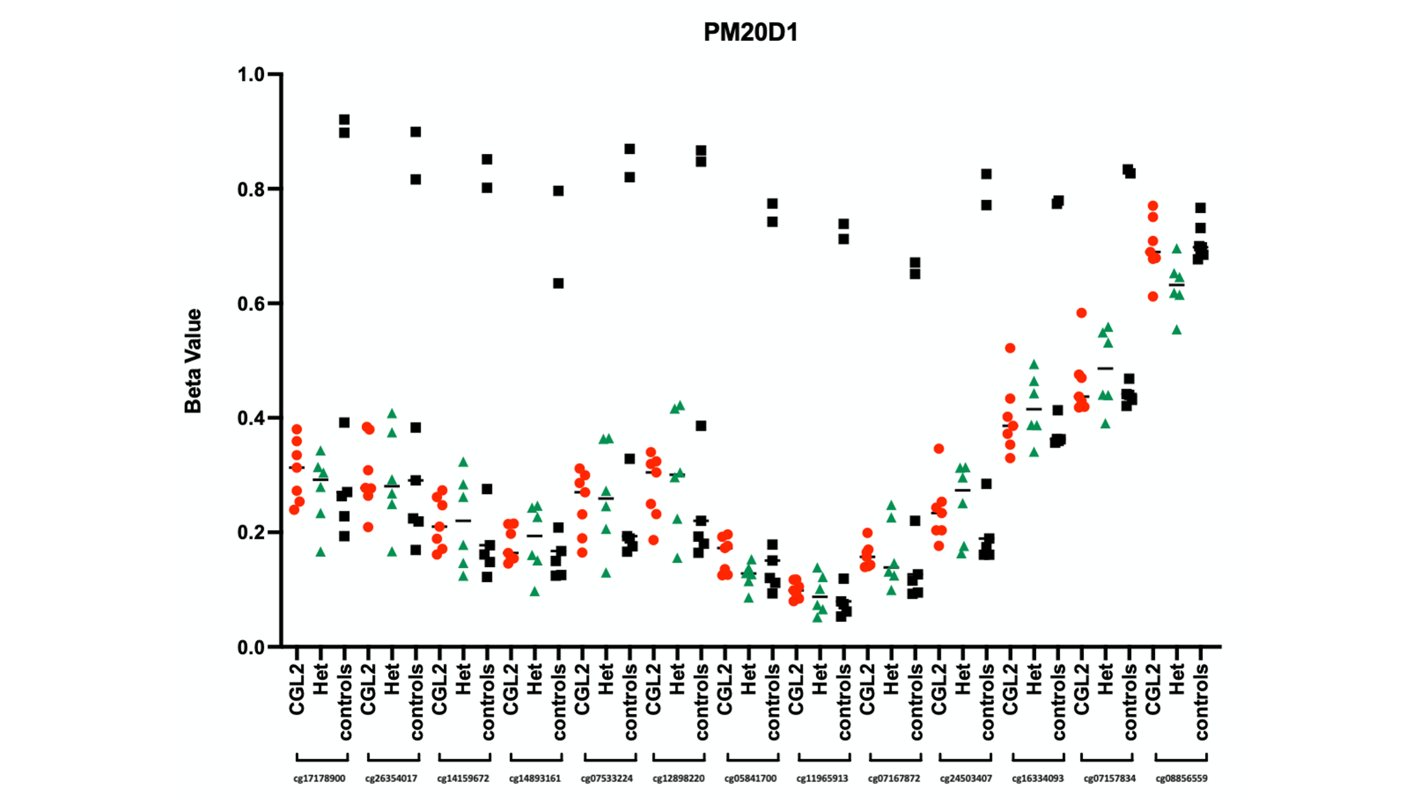


**Supplementary Figure 5.** DNA methylation of the differentially methylated sites in the *PM20D1* promoter

**
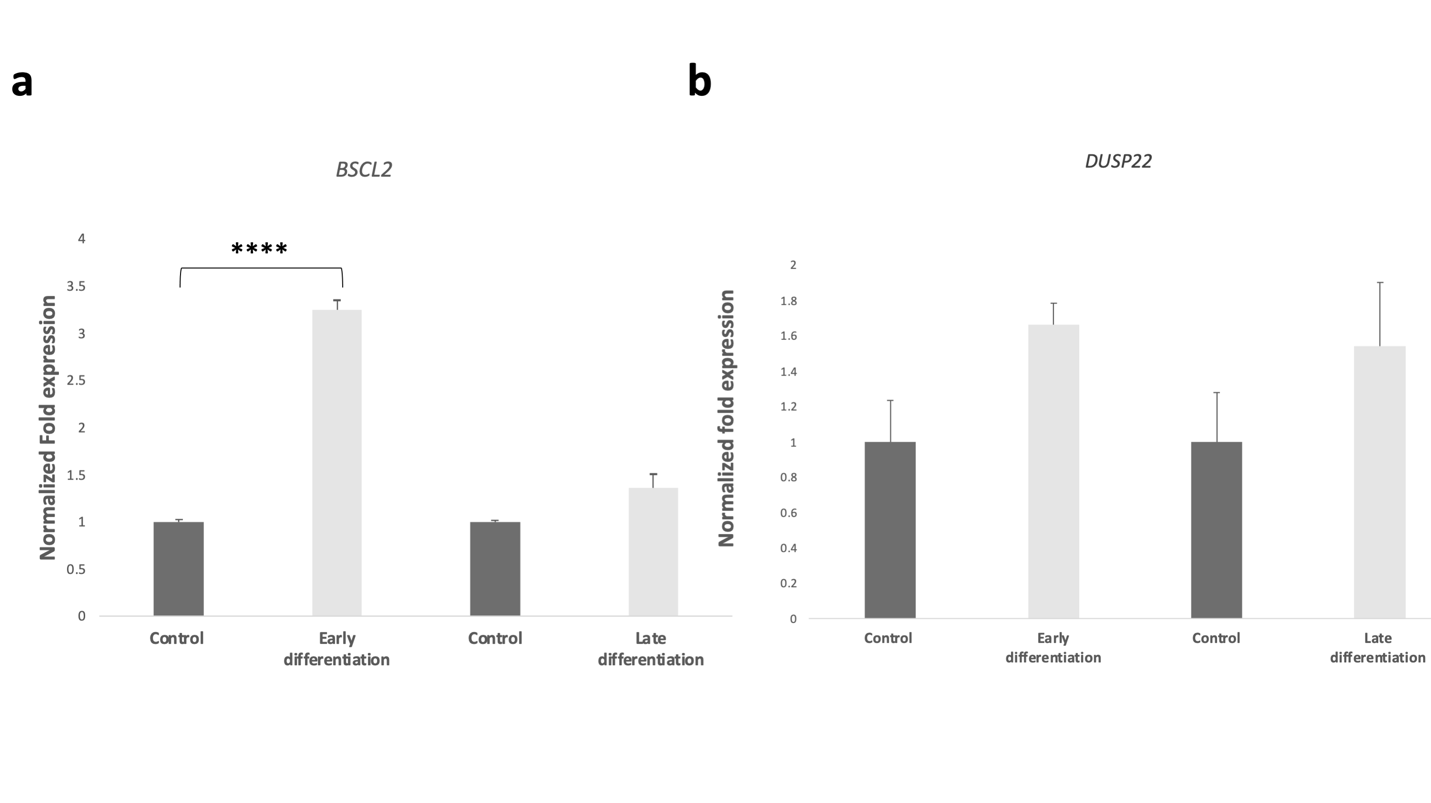
Supplementary Figure 6 a.** *BSCL2* and **b.** *DUSP22* gene expression changes during early and late adipocyte differentiation.


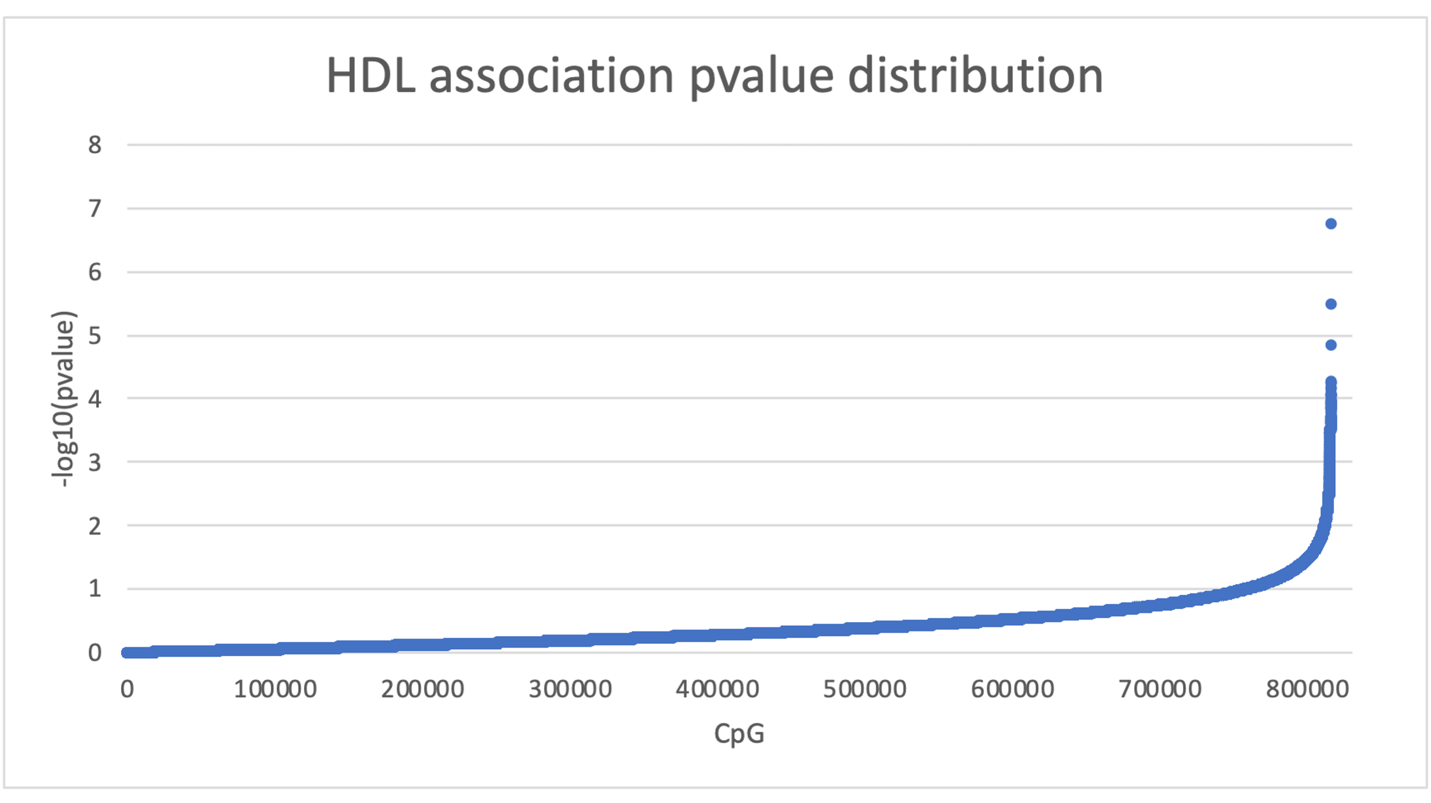


**Supplementary Figure 7.** DNA methylation association of CpG sites in the *DUSP22* promoter with HDL in an EPIC methylation array dataset of 568 healthy controls from the Qatar BioBank.


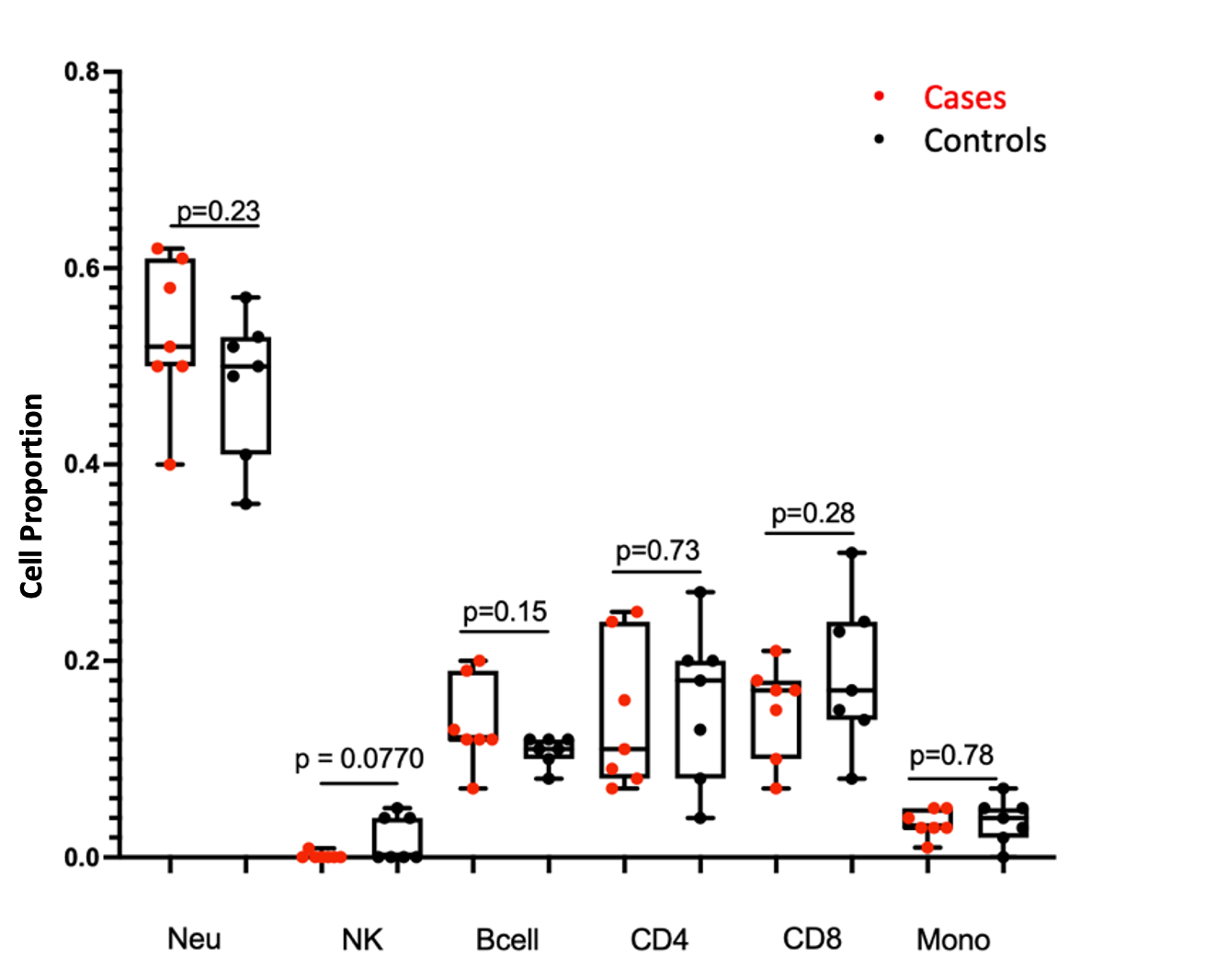


**Supplementary Figure 8.** Comparison of deconvoluted cell proportions measured via the Houseman method between cases and controls
